# Baricitinib Attenuates Autoimmune Phenotype and Podocyte Injury in a Murine Model of Systemic Lupus Erythematosus

**DOI:** 10.3389/fimmu.2021.704526

**Published:** 2021-08-23

**Authors:** Jaeseon Lee, Youngjae Park, Se Gwang Jang, Seung-Min Hong, Young-Seok Song, Min-Jun Kim, SeungYe Baek, Sung-Hwan Park, Seung-Ki Kwok

**Affiliations:** ^1^The Rheumatism Research Center, Catholic Research Institute of Medical Science, College of Medicine, The Catholic University of Korea, Seoul, South Korea; ^2^Division of Rheumatology, Department of Internal Medicine, Seoul St. Mary’s Hospital, College of Medicine, The Catholic University of Korea, Seoul, South Korea; ^3^Department of Biomedicine & Health Sciences, College of Medicine, The Catholic University of Korea, Seoul, South Korea

**Keywords:** systemic lupus erythematosus, baricitinib, renal inflammation, podocyte, B cell

## Abstract

**Objective:**

Baricitinib, a selective inhibitor for janus kinase (JAK) 1 and JAK2, is approved for use in rheumatoid arthritis. Systemic lupus erythematosus (SLE) is recently regarded as a potential candidate targeted by JAK inhibitors because of the relationship between its pathogenesis and JAK/signal transducer and activator of transcription (STAT) pathway-mediated cytokines such as type I interferons. The objective of this study was to determine whether baricitinib could effectively ameliorate SLE using a murine model

**Methods:**

To investigate effects of baricitinib on various autoimmune features, especially renal involvements in SLE, eight-week-old MRL/Mp-*Fas^lpr^* (MRL/*lpr*) mice were used as a lupus-prone animal model and treated with baricitinib for eight weeks. Immortalized podocytes and primary podocytes and B cells isolated from C57BL/6 mice were used to determine the *in vitro* efficacy of baricitinib.

**Results:**

Baricitinib remarkably suppressed lupus-like phenotypes of MRL/*lpr* mice, such as splenomegaly, lymphadenopathy, proteinuria, and systemic autoimmunity including circulating autoantibodies and pro-inflammatory cytokines. It also modulated immune cell populations and effectively ameliorated renal inflammation, leading to the recovery of the expression of structural proteins in podocytes. According to *in vitro* experiments, baricitinib treatment could mitigate B cell differentiation and restore disrupted cytoskeletal structures of podocytes under inflammatory stimulation by blocking the JAK/STAT pathway.

**Conclusions:**

The present study demonstrated that baricitinib could effectively attenuate autoimmune features including renal inflammation of lupus-prone mice by suppressing aberrant B cell activation and podocyte abnormalities. Thus, baricitinib as a selective JAK inhibitor could be a promising therapeutic candidate in the treatment of SLE.

## Introduction

The janus kinase (JAK)/signal transducer and activator of transcription (STAT) pathway is known to be a hub of intracellular signal cascade for more than 50 cytokines to bind to type I and type II cytokine receptors (1). Four JAKs (JAK1–3, and TYK2) and seven STAT proteins (STAT1–4, STAT5A, STAT5B, and STAT6) can form homo- and hetero-dimers, make pairs with each other, and transduce cellular signals (1). As the relationship between specific cytokines and inflammatory diseases has been discovered, the JAK/STAT pathway has also been focused as a therapeutic target. Various small molecules as JAK inhibitors have been developed to block phosphorylation of JAKs (2). They are being investigated in clinical trials for autoimmune inflammatory diseases such as ankylosing spondylitis, psoriatic arthritis, and inflammatory bowel diseases (3). Some early generations of JAK inhibitors are currently approved for use in the treatment of rheumatoid arthritis.

Systemic lupus erythematosus (SLE) is a prototypic autoimmune disease mainly affecting young women with multiple organ involvements, including the skin, joints, central nervous systems, and kidneys (4). Although formation and deposition of immune complexes and consequent activation and infiltration of various immune cells into targeted tissues are regarded as main mechanisms of immunopathogenesis in SLE (4, 5), disease-curing agents for SLE have not been discovered yet. Several decades ago, type I interferons (IFNs) were suggested as potential contributors to the pathophysiology of SLE by inducing differentiation, maturation and activation of autoreactive T cells, B cells, and other monocytes (6–8). Therefore, multiple pharmacologic agents targeting IFN-related pathways have been attempted to treat SLE. Although, most novel treatments have not met goals of primary outcomes in clinical trials (9), JAK inhibitors that could suppress type I and II IFNs and other cytokines involved in SLE have shown some promising results *in vitro*, *in vivo*, and in human studies (10).

Indeed, tofacitinib, the first generation JAK inhibitor, can mitigate lupus-like features and serological parameters indicating the degree of autoimmunity in multiple mice models, although results involving human data are insufficient (11). Baricitinib is a more recently approved JAK inhibitor. A phase 2 trial for baricitinib in patients with non-renal involvements have been finished with positive results (12). Baricitinib is a selective JAK1 and JAK2 inhibitor. It is expected to demonstrate efficacy for SLE by blocking signal pathways related to type I and II IFNs and pro-inflammatory cytokines such as interleukin (IL)-6 (13). Despite its therapeutic potentials for managing various manifestations of SLE, clinical trials and several case reports of baricitinib have been confined to cutaneous and articular involvements, excluding subjects with lupus nephritis (LN), one of the most significant organ involvements in SLE (12, 14, 15). In addition, pre-clinical evidences delineating the potential efficacy of baricitinib on lupus-related phenotypes and its mechanism remain to be determined.

To investigate effects of baricitinib on autoimmunity and renal phenotypes of SLE, MRL/Mp-*Fas^lpr^* (MRL/*lpr*) mice were selected as an animal model recapitulating various clinical features of SLE. Herein, we demonstrate that baricitinib can effectively ameliorate lupus-mimicking features including renal inflammation, serological profiles, and proportions of pathogenic immune cells in a lupus-prone murine model. In addition, we found that the improvement of overall autoimmunity and renal function was attributable to its pharmacological inhibition of the JAK/STAT pathway in B cells and podocytes by suppressing their differentiation and stabilizing cellular cytoskeleton, respectively.

## Materials and Methods

### Animals and Treatment

Female MRL/*lpr* mice were purchased from SLC Inc (Japan). These animals were maintained under specific pathogen-free conditions from 7–16 weeks of age. C57BL/6 mice were purchased from OrientBio (Korea). All procedures of animal research were performed in accordance with the Laboratory Animals Welfare Act, the Guide for the Care and Use of Laboratory Animals and the Guidelines and Policies for Rodent experiment provided by the Institutional Animal Care and Use Committee (IACUC) of the School of Medicine, The Catholic University of Korea (approval numbers: CUMS-2018-0341-01 and 2018-0236-01). Baricitinib (MyBioSource) was administered to MRL/*lpr* mice twice daily at 10 mg/kg in 0.5% methyl cellulose (Sigma) from 8 weeks of age for a total of 8 weeks. Body weights were checked every two weeks.

### Enzyme-Linked Immunosorbent Assay (ELISA)

Mice sera were diluted at 1:100,000 for immunoglobulin (Ig) G, 1:50,000 for IgG2a, 1:1,000 for anti-double strand DNA (dsDNA) IgG, 1:10 for IL-6 and IL-17, and 1:20 for B cell activating factor (BAFF) respectively. Mouse total IgG quantitation set and mouse total IgG2a quantitation set were acquired from Bethyl laboratory. Mouse anti-dsDNA IgG quantitation kit was purchased from Alpha Diagnostics. Mouse IL-6 ELISA Duoset, mouse IL-17 ELISA Duoset, and mouse BAFF ELISA Duoset were obtained from R&D systems. Albumin and creatinine levels in urine samples of mice at 16 weeks of age were analyzed using mouse albumin ELISA set (Bethyl lab) and creatinine parameter assay kit (R&D systems), respectively. All tests were performed following the manufacturer’s instructions.

### Flow Cytometry

Spleens and kidneys were minced and filtered through a 40-μm cell strainer (Falcon) to prepare single-cell suspensions. Red blood cells (RBCs) were removed with ammonium-chloride-potassium (ACK) lysis buffer. For intracellular staining, cells were stimulated with 25 ng/mL phorbol 12-myristate 13-acetate (PMA, Sigma) and 250 ng/mL ionomycin (Sigma) with monensin-containing Golgistop (BD Biosciences) for five hours. Cells were harvested and stained with a eFluor 780-Fixable viability dye (eBioscience) to exclude dead cells for analysis. Cells were stained with Pacific blue anti-CD90.2, PerCP-Cy5.5 anti-CD4, PE anti-CD8a, APC anti-CD19, FITC anti-CXCR5, BV605 anti-CD279 (PD-1), BV605 anti-CD44, Alexa 488 anti-CD62L (Biolegend), APC anti-CD25, PE anti-Foxp3 antibodies (eBioscience), and PE anti-CD138 antibodies (BD Biosciences). Flow cytometric analysis was performed using BD LSRFortessa (BD Biosciences). Data were analyzed using BD FACSDiva (version 10, BD Biosciences).

### Renal Histology and Immunohistochemical Staining

Mice were anesthetized using a zoletil-xylazine cocktail during perfusion with phosphate-buffered saline (PBS). Kidney was vertically dissected and fixed with 10% formalin. Kidney sections (3 μm in thickness) were stained with Periodic acid-Schiff (PAS) stain (Sigma). Kidney pathology was evaluated using a lupus nephritis classification system as described in a previous study (16). Immunohistochemistry was performed using a Vectastain ABC kit (Vector). Tissue sections were incubated with anti-nephrin (Progen), anti-synaptopodin (Abcam), anti-podocin (Abcam), or isotype control antibodies (Abcam) at 4°C overnight. Staining was developed using 3,3′-diaminobenzidine chromogen (Dako). Sections were counterstained with hematoxylin QS (Vector).

### Confocal Microscope

Tissue sections were stained with antibodies to IgG (Bethyl lab) and nephrin (Abcam) for 16 hours at 4°C followed by staining with Alexa594 conjugated anti-rabbit IgG, Alexa488 conjugated anti-guinea pig IgG, and Alex594 conjugated anti-phalloidin (Invitrogen). Nuclei were stained with 4’, 6-diamidino-2-phenylindole dihydrochloride (DAPI, Invitrogen). Isotype-control staining was conducted *via* probing with rabbit IgG rather than with primary antibodies. Confocal images were acquired using an LSM 700 confocal microscope (Zeiss).

### Splenocyte *Culture*

Mice spleens were removed and prepared for single cell suspension using glass teasing slides and a 40-μm cell strainer (BD Biosciences). RBCs were lysed with ACK lysis buffer (Thermo). Splenocytes were cultured with RPMI1640 media (Gibco) supplemented with 10% fetal bovine serum (AB Frontier).

### B Cell Differentiation

CD19+ B cells from C57BL/6 mice and MRL/*lpr* mice were obtained by magnetic activated cell sorting using CD19 microbeads (Miltenyi Biotec). The purity was over 95% as demonstrated by flowcytometry. CD19+ B cells were stimulated with 1 μg/mL F(ab′)2-goat anti-mouse IgM antibodies (eBioscience), 250 μg/mL soluble CD40 ligand (sCD40L) and 100 ng/mL recombinant mouse IL-4 (Peprotech) for 2 or 5 days with or without baricitinib pre-treatment. RPMI1640 medium supplemented with a 10% FBS was used for their culture.

### Isolation of Glomeruli and Podocyte Culture

Primary mouse glomeruli were harvested using magnetic isolation protocols. Briefly, C57BL/6 mice that had received Dynabead M-450 (Invitrogen) perfusions were euthanized. Their kidneys were dissected and digested with type I collagenase (Gibco) for 30 minutes at 37°C. They were then passed through a 100-μm cell strainer. The supernatant was discarded and the cell pellet was resuspended in 2 mL of Hanks balanced salt solution (HBSS, Gibco). Dynabead-trapped glomeruli were isolated using a magnetic particle concentrator and washed with HBSS. For podocyte primary culture, freshly isolated glomeruli were plated onto 60 mm dishes coated with collagen at 37°C in DMEM/F12 (Gibco) supplemented with 5% fetal bovine serum (Gibco), 0.5% Insulin-Transferrin-Selenium liquid (Gibco), and 1% penicillin–streptomycin (Gibco). The outgrowth of podocytes started between day 2 and 3. Seven days later, glomeruli and outgrowth cells were detached from the plate using a 0.25% trypsin-EDTA solution (Gibco). Trypsinized cells were strained by passing a 40-mm cell strainer and plated onto collagen-coated dishes. Podocytes of passages 1 were used in all experiments. Mouse podocyte cell line E11 was acquired from Cell Line Service (Germany). These cells were maintained with RPMI1640 media supplemented with 10% FBS at 33°C in a humidified atmosphere with 5% CO2. To differentiate podocytes, cells were transferred to a non-permissive condition at 37°C for 12–14 days.

### Quantitative Polymerase Chain Reaction (qPCR)

Total RNA was collected using an RNAiso Plus reagent (Takara). Up to 1–2 µg of total RNA was converted to complementary DNA (cDNA), using a Transcriptor First-Strand cDNA Synthesis kit (Roche). A LightCycler 96 instrument (Roche) was used for PCR amplification and analysis. All reactions were performed with SYBR Green I Master, according to the manufacturer’s instructions. Primers were designed with a web tool from GenScript (http://www.genscript.com). Primer sequences are as follows (forward and reverse, respectively): *Actb* (beta-actin), 5′ - GAAATCGTGCGTGACATCAAAG - 3′ and 5′ - TGTAGTTTCATGGATGCCACAG - 3′; *Gapdh*, 5′ - CAGCAACTCCCACTCTTCCAC - 3′ and 5′ - TGGTCCAGGGTTTCTTACTC - 3′; *Nphs1* (nephrin), 5′ - ACTACGCCCTCTTCAAATGCA - 3′ and 5′ - TCGAGGGCCTCATACCTGAT - 3′; *Synpo* (synaptopodin), 5′ - TATCAACCAGAACCCGTC - 3′ and 5′ - AATCAAGTGTGCCATTCG - 3′; *Nphs2* (podocin), 5′ - CCATCTGGTTCTGCATAAAGG - 3′ and 5′ - CCAGGACCTTTGGCTCTTC - 3′; *Aicda*, 5′ - CGTGGTGAAGAGGAGAGATA - 3′ and 5′ - CAGTCTGAGATGTAGCGTAG -3′; *Bcl6*, 5′ - CCCTGTGAAATCTGTGGCAC - 3′ and 5′ - ACACGCGGTATTGCACCTTG - 3′; *Xbp1*, 5′ - GCAAGTGGTGGATTTGGAAG - 3′ and 5′ - CCTCTGGAACCTCGTCAG - 3′; *Irf4*, 5′ - ACCAGTCACACCCAGAAATC - 3′ and 5′ - GGGCACAAGCATAAAAGGTTC - 3′; *Ifit1*, 5′ - CTGAAATGCCAAGTAGCAAGG - 3′ and 5′ - CCAAAGGCACAGACATAAGGA - 3′; *Isg15*, 5′ - AGTGCTCCAGGACGGTCTTA - 3′ and 5′ - TCGCTGCAGTTCTGTACCAC - 3′; *Mx1*, 5′ - GATCCGACTTCACTTCCAGA -3′ and 5′ - CATCTCAGTGGTAGTCAACC- 3′. All mRNA expression levels were normalized to the expression of *Actb* or *Gapdh*. Relative fold induction was calculated using 2 - (ΔCq) or 2 - (ΔΔCq), where ΔΔCq was ΔCq (stimulated) - ΔCq(unstimulated), ΔCq was Cq (target) - Cq (*Actb* or *Gapdh*), and Cq was the cycle at which the threshold was crossed. PCR product quality was monitored using post-PCR melting curve analysis.

### Western Blot

Cytosolic proteins were extracted using RIPA buffer containing Halt protease/phosphatase inhibitor cocktail (Thermo). Membrane fraction of cell lysates were extracted using Mem-PER Plus membrane protein extraction kit (Thermo). For immunoblotting, proteins (15–30 μg for each sample) were separated by 10–12% sodium dodecyl sulfate polyacrylamide gel electrophoresis (SDS-PAGE), transferred onto polyvinylidene fluoride membranes (Biorad) and probed with the following antibodies: anti-pSTAT1_Y701_, anti-STAT1, anti-pSTAT3_Y705_, anti-pSTAT3_S727_, anti-STAT3, anti-p-JAK2, anti-JAK2 (Cell signaling technology), anti-nephrin (Progen), anti-podocin, anti-Na/K ATPase (Abcam) and anti-β-actin (Sigma). Subsequently, membranes were incubated with horseradish peroxidase-conjugated goat anti-rabbit IgG or goat anti-mouse IgG (Thermo). Reactive proteins on membranes were visualized using a SuperSignal West Pico Chemiluminescent substrate (Thermo). These membranes were then exposed to an Amersham Imager 600 (GE Healthcare).

### Statistics

All statistical analyses were performed using GraphPad Prism (version 8, GraphPad Software). Statistical significance was determined by the Mann Whitney test, or by one-way analysis of variance with Tukey multiple comparison test. Statistically significant difference was considered when *p* value was less 0.05.

## Results

### *In Vivo* Treatment With Baricitinib Abrogates Lupus-Like Phenotype of MRL/lpr Mice

**Figure 1:**
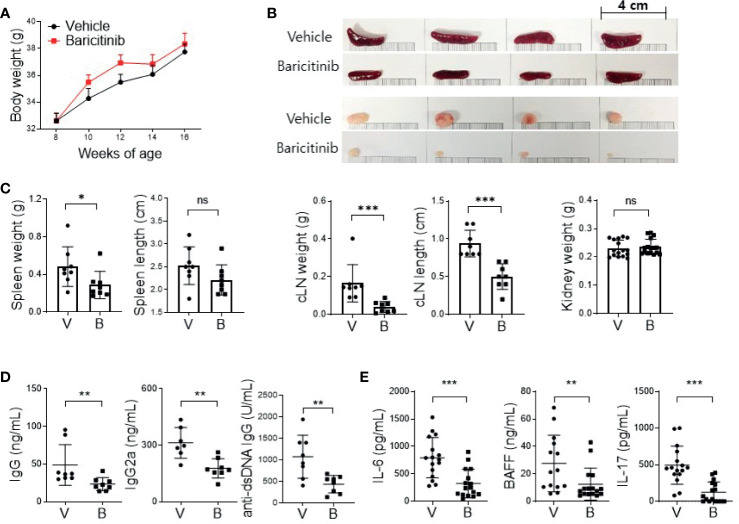
*In vivo* treatment with baricitinib abrogates lupus-like phenotype of MRL/*lpr* mice. Eight-week-old MRL/*lpr* mice (n = 8 in each group) were treated by gavage with either vehicle (V) only or baricitinib (B, 10 mg/kg) twice daily for eight weeks. Mice were euthanized at the age of 16 weeks. **(A)** Body weights of mice from each group are shown. **(B)** Representative images showing gross appearance of spleens (the upper panels) and cervical lymph nodes (cLNs, the lower panels) from the two groups of mice. **(C)** Weights of spleen, cLN, and kidney with length of spleen and cLN from each group of mice are shown. **(D)** Serum levels of IgG, IgG2a, and anti-dsDNA in the two group of mice were determined by ELISA. **(E)** Serum levels of pathogenic cytokines including IL-6, BAFF and IL-17 were measured by ELISA. Data are expressed as mean ± SD. *p < 0.05; **p < 0.01; ***p < 0.001; ns, not significant.

MRL/*lpr* mice presented lupus-like features such as organomegaly and renal inflammation with seropositivity of multiple autoantibodies from the age of 12 weeks (17). To determine whether baricitinib could suppress lupus-mimicking phenotypes in these mice, we administered vehicles with or without baricitinib to 8-week-old MRL/*lpr* mice for 8 weeks. Baricitinib-treated mice showed higher body weights during *in vivo* experiments (**Figure 1A**) from 8-week-old to 16-week-old. Baricitinib treatment resulted in lower degrees of splenomegaly and lymphadenopathies at the age of 16 weeks (**Figure 1B** and **Supplementary Figure 1**) compared to control (vehicle treatment). Weights of spleens and cervical lymph nodes from baricitinib-treated mice were significantly lower than those of controls (**Figure 1C**). Circulating IgG, IgG2a, and anti-dsDNA IgG levels measured from sera of mice were significantly lower in the baricitinib-treated group than in the vehicle control group (**Figure 1D**). Serum levels of lupus-related pro-inflammatory cytokines including IL-6, BAFF, and IL-17 were markedly decreased by baricitinib treatment (**Figure 1E**). These results suggest that the administration of baricitinib to MRL/*lpr* mice can ameliorate the lupus-like phenotypes by suppressing systemic autoimmunity.

### Effects of *In Vivo* Treatment With Baricitinib on Subsets of T Cells in MRL/lpr Mice

**Figure 2:**
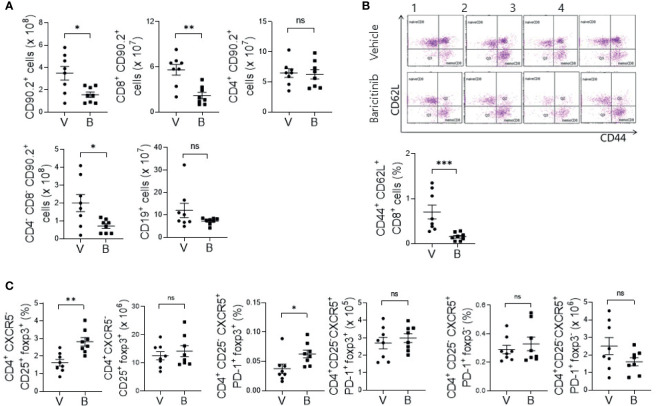
Effects of *in vivo* treatment with baricitinib on subsets of T and B cells in spleens of MRL/*lpr* mice at the age of 16 weeks. Subsets of T and B cells from each group of mice were determined using flow cytometry. **(A)** Absolute cell numbers of total T cells (CD90.2+ cells), CD8+ T cells, CD4+ T cells, double negative T (DNT) cells (CD4-CD8-CD90.2+ T cells), and total B cells (CD19+ cells) are shown. **(B)** Representative flow cytometric plots of central memory CD8+ T cells (CD44+CD62L+CD8+ cells) are shown in the upper panel. The percentage of central memory CD8+ T cells among total T cells is shown in the lower panel. **(C)** The absolute numbers and percentages of CD4+CD25+CXCR5-FOXP3+ cells (Treg cells), CD4+CD25-CXCR5+PD-1+FOXP3+ cells (Tfr cells), and CD4+CD25-CXCR5+PD-1+FOXP3- (Tfh cells) among total T cells are shown. Data are expressed as mean ± SD. *p < 0.05; **p < 0.01; ***p < 0.001; ns, not significant.

MRL/*lpr* mice with systemic autoimmune problem showed abnormalities of T and B cells, resembling human SLE (18). Using flow cytometric analysis, we measured absolute counts and proportions of T cell and B cell subsets among splenocytes from baricinitib-treated or vehicle-only-treated mice. Total T cells (CD90.2+) and CD8+ T cells were significantly decreased in 16-week-old mice treated with baricitinib than in control mice treated with the vehicle (**Figure 2A**). DNT cells (CD4-CD8-) are related to SLE pathogenesis and activities of lupus-prone mice and SLE patients (19, 20). Their numbers increased according to the disease severity in this mouse model. Baricitinib reduced the numbers of DNT cells in MRL/*lpr* mice at the age with the disease fully blown whereas total B cells (CD19+) were not influenced. Intriguingly, among various T cell subsets, central memory CD8+ T cells (CD44+CD62L+, **Supplementary Figure 2**) were the most remarkably reduced subpopulation after baricitinib treatment (**Figure 2B**). Their roles have not been fully defined in the pathogenesis of SLE. Furthermore, the proportion of regulatory T (Treg) cells (CD4+CXCR5-CD25+FOXP3+) and follicular regulatory T (Tfr) cells, which can modulate germinal center (GC) reactions, were significantly increased in splenocytes from baricitinib-treated mice while the absolute numbers of Treg cells and Tfr cells, and both the absolute numbers and the proportion of follicular helper T (Tfh) cells showed no significant differences (**Figure 2C** and **Supplementary Figure 3**) after baricitinib treatment. Collectively, these results indicate that baricitinib can modulate abnormal expansion of lupus-pathogenic T cell populations by reducing DNT cells while increasing Treg and Tfr cells.

### Baricitinib Ameliorates Autoimmune Kidney Damage in MRL/lpr Mice

**Figure 3:**
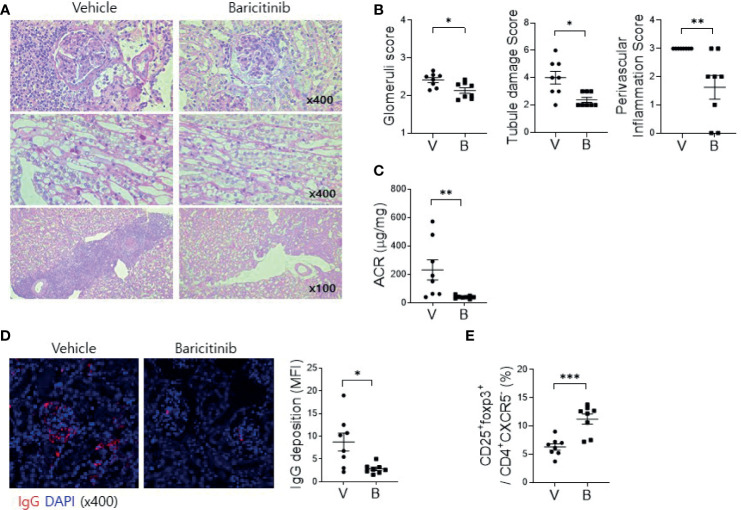
Baricitinib ameliorates autoimmune kidney damage in MRL/*lpr* mice. **(A)** Representative images of PAS staining of kidney sections from each group of MRL/*lpr* mice treated with baricitinib (B) or vehicle (V), showing glomerular pathology (top), tubular damage (middle), and perivascular cell accumulation (bottom). **(B)** Glomerular pathology score, tubular damage score, and the degree of perivascular cell accumulation are shown. **(C)** Urinary albumin and creatinine (Cr) levels from each group of mice at the age of 16 weeks were measured by ELISA. Albumin/Cr ratio (ACR) is shown. **(D)** Representative confocal microscopic images of kidney sections from MRL/*lpr* mice. Tissue sections were stained with fluorescence-conjugated antibodies to IgG (red) (original magnification ×400). IgG-positive area was calculated using ImageJ software. Mean fluorescence intensity (MFI) are presented (right panel). **(E)** Flow cytometric analysis showing populations of CD4+CD25+CXCR5-FOXP3+ cells (Treg cells) in kidney tissues from MRL/lpr mice. The percentage of Treg cell population among total lymphocytes is shown. Data are expressed as mean ± SD. *p < 0.05; **p < 0.01; ***p < 0.001.

Deposition of immune complex and in-situ infiltration of various immune cells can cause profound inflammation in renal tissues of MRL/*lpr* mice. Such renal inflammation can also occur in SLE patients, frequently resulting in compromised renal function. Therefore, renal inflammation is regarded as the main cause of mortality of patients with SLE. The objective of this study was to evaluate whether administration of baricitinib could reduce renal inflammation in a lupus-animal model. Kidney tissues were acquired from 16-week-old MRL/*lpr* mice with or without baricitinib treatment. They were then assessed for the degree of inflammation. Histopathologic findings showed markedly reduced inflammation in the glomerulus, tubules, and perivascular area of kidney tissue sections from baricitinib-treated mice (**Figures 3A, B**). Urine albumin/creatinine ratio was also remarkably decreased in mice treated with baricitinib, indicating reduced proteinuria and renal damages (**Figure 3C**). Confocal microscopic images demonstrated decreases of IgG deposition in renal tissues from baricitinib-treated mice compared to control (vehicle-treated) mice (**Figure 3D**). In addition, Treg cells in murine kidneys were increased by baricitinib treatment (**Figure 3E** and **Supplementary Figure 4**).

### Baricitinib Increases the Expression of Functional Molecules of Podocyte

**Figure 4:**
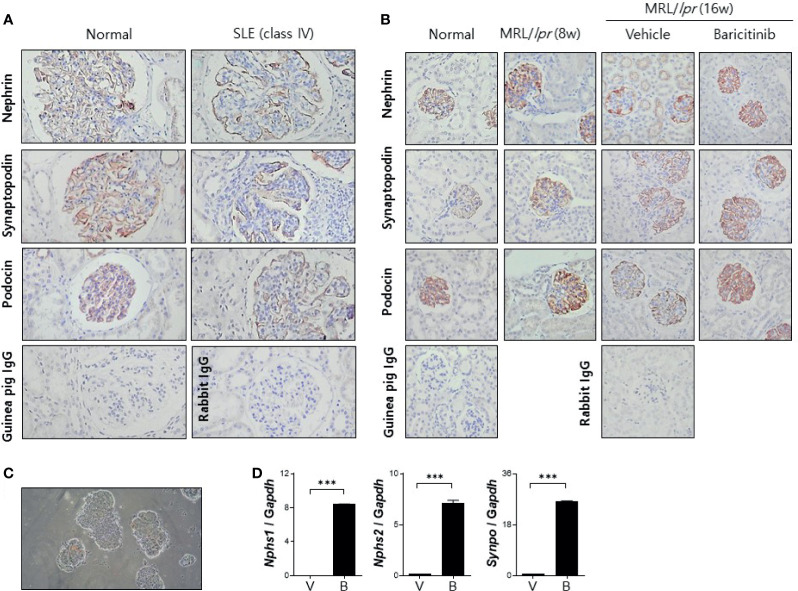
Baricitinib increases expression levels of functional membranous molecules of podocytes including nephrin, synaptopodin, and podocin in MRL/*lpr* mice. **(A)** Representative pictures of immunohistochemical staining of nephrin, synaptopodin, and podocin in human kidneys (original magnification ×100) (normal control: left panel; a female patient with lupus nephritis ISN/RPS class IV: right panel). Expression levels of membranous molecules of podocytes were decreased in the glomeruli of patients with lupus nephritis. **(B)** *In vivo* treatment with baricitinib increases the expression of nephrin, synaptopodin and podocin in kidneys of 16-week-old MRL/*lpr* mice. Representative images of immunohistochemical staining of nephrin, synaptopodin, and podocin in kidneys of 16-week-old MRL/MpJ mice (normal controls), 8-week-old MRL/*lpr* mice, and 16-week-old MRL/*lpr* mice treated with vehicle or baricitinib are shown (original magnification ×400). **(C)** Representative images of glomeruli isolated from 16-week-old MRL/*lpr* mice are shown (original magnification ×400). **(D)** mRNA levels of *Nphs1* (nephrin), *Nphs2* (podocin), and *Synpo* (synaptopodin) from pooled glomeruli of eight mice of each group were determined by real-time PCR. Data are expressed as mean ± SD. ***p < 0.001.

Podocytes are specialized epithelial cells that can maintain selective filtering functions of renal glomerulus due to their unique morphologic features known as foot processes and slit diaphragms. Renal inflammation in patients with LN can result in decreased expression of functional proteins in podocytes, thereby affecting the structural integrities of these specialized cellular barriers (21). We investigated whether baricitinib could restore podocyte functions by increasing impaired expression of intra- and extra-cellular key proteins in podocytes of lupus-prone mice. At first, decreased expression levels of nephrin and podocin as elemental proteins of slit diaphragm and synaptopodin as the main regulator of actin cytoskeleton of foot process in glomeruli from patients with LN were confirmed by comparing with healthy controls (**Figure 4A**). We then assessed expression levels of these functional proteins in podocytes from MRL/*lpr* mice treated with or without baricitinib. Immunohistological results demonstrated that all podocyte-related proteins expressed in renal glomeruli from baricitinib-treated mice at the age of 16 weeks were remarkably increased (**Figure 4B**). Expression levels of mRNA for nephrin (*Nphs1*), podocin (*Nphs2*) and synaptopodin (*Synpo*) measured from isolated glomeruli (**Figure 4C**) of baricitinib-treated mice were also significantly elevated than those from the vehicle-only-treated mice (**Figure 4D**). All these results indicate that baricitinib has therapeutic effects on renal inflammation in MRL/*lpr* mice. In addition, improved SLE features of mice after treatment with baricitinib were due to its ability to restore functions of specialized glomerular cells, podocytes, in targeted tissues.

### Baricitinib Suppresses B Cell Differentiation and IgG Production *In Vitro*

**Figure 5:**
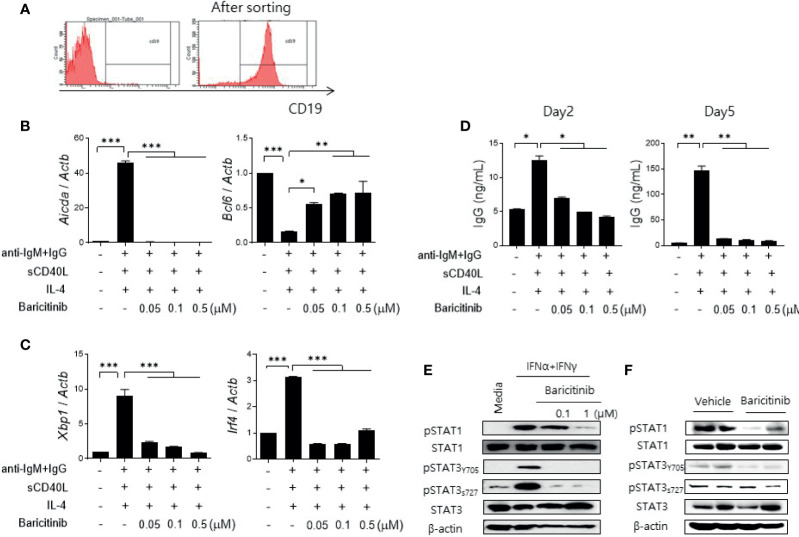
Baricitinib suppresses B cell differentiation and IgG production *in vitro*. **(A)** Representative flow cytometric image showing CD19+ B cells isolated from spleens of C57BL/6 mice. **(B)** B cells (5 × 10^5^ cells per well) treated with 1 μg/mL IgM antibodies, 250 ng/mL sCD40L, and 100 ng/mL IL-4 for 2 days with or without graded dose of baricitinib. mRNA levels of *Aicda*, and *Bcl6* were determined using real-time PCR. **(C)** B cells were treated with the same condition for 5 days. mRNA levels of *Xbp1* and *Irf4* were determined using real-time PCR. **(D)** Levels of IgG in culture supernatant were measured by ELISA. **(E)** CD19+ B cells treated with 100 U/mL IFN-α and 20 ng/mL IFN-γ for 30 minutes with or without baricitinib. Expression levels of pSTAT1, STAT1, pSTAT3 (Tyr705, Ser727), and STAT3 are shown. **(F)** Expression levels of pSTAT, STAT1, pSTAT3 (Tyr705, Ser727), and STAT3 in CD19+ B cells from 16-week MRL/*lpr* mice *in vivo* treated with baricitinib or vehicle. These western blot data are representatives of three independent experiments. Bars indicate mean ± SD. *p < 0.05; **p < 0.01; ***p < 0.001.

Cytokines related to B cell differentiation and maturation exert their functions mainly *via* the JAK/STAT pathway (13, 22, 23). Therefore, we determined whether the administration of baricitinib could repress the expression of genes related to differentiation of naïve B cells. CD19+ B cells were isolated from spleens of C57BL/6 mice (**Figures 5A–D**) and MRL/*lpr* mice (**Supplementary Figure 5**) using microbeads. Their purity was validated by flow cytometric analysis (**Figure 5A**). These cells were treated by graded doses of baricitinib and stimulated under the same conditions, inducing B cell maturation with anti-mouse IgM antibodies, sCD40L, and IL-4 for 2 or 5 days, respectively. After two days of stimulation, the expression level of *Aicda* was found to be significantly suppressed by baricitinib in a dose-dependent manner, whereas that of *Bcl6* was increased (**Figure 5B**). Expression levels of *Xbp1* and *Irf4* were also remarkably decreased after 5 days of stimulation (**Figure 5C**). In addition, the amount of IgG production measured from supernatants under the same conditions was significantly reduced depending on increasing doses of baricitinib after both two days and five days of stimulation, respectively (**Figure 5D**). The similar tendency was also replicated in the results of the *in vitro* experiments using B cells from MRL/*lpr* mice (**Supplementary Figure 5**).

Blockade of the JAK/STAT pathway in B cells was assessed by measuring expression levels of phosphorylated STAT proteins using immunoblot. Baricitinib treatment effectively suppressed the expression of phosphorylated STAT1 (pSTAT1) and STAT3 (pSTAT3), both tyrosine 705 (pSTAT3_Y705_) and serine 727 (pSTAT3_S727_) of pSTAT3, respectively, in naïve B cells from C57BL/6 mice after stimulation with type I and II IFNs (**Figure 5E**). These results were consistent with results using CD19+ B cells acquired from splenocytes of MRL/*lpr* mice at the age of 16 weeks and treated with the same manner as previously described (**Figure 5F**). Overall, baricitinib effectively repressed signals of the JAK/STAT pathway in B cells both *in vitro* and *in vivo*. These effects on naïve B cells could result in suppression of B cell differentiation and immunoglobulin production that are critical for the pathogenesis of systemic autoimmunity in SLE.

### Baricitinib Stabilizes Podocyte Cytoskeleton

**Figure 6:**
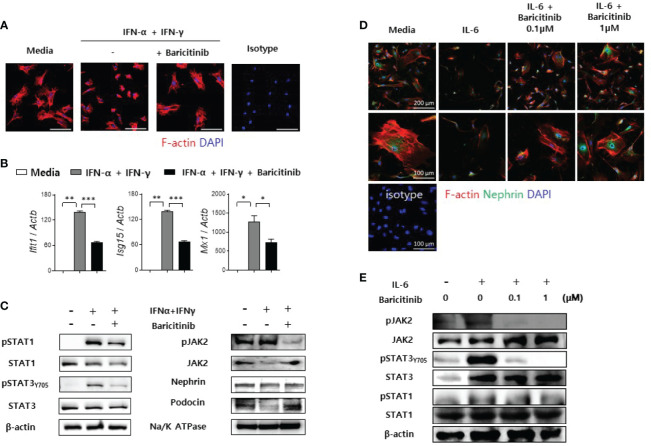
Baricitinib stabilizes podocyte cytoskeleton. **(A)** After podocyte cell line was differentiated in 37°C permissive condition over 14 days, it was stimulated by 100 U/mL IFN-α and 20 ng/mL IFN-γ for 2 days with or without 1 μM baricitinib. Representative confocal microscopic images show actin cytoskeleton of podocytes (phalloidin staining, red). **(B, C)** Differentiated podocytes were stimulated by the same condition for 4 hours **(B)** or 30 minutes **(C)**. mRNA levels of *Ifit1*, *Isg15*, and *Mx1* were determined using real-time PCR **(B)**. Expression levels of pJAK2, JAK2, pSTAT1, STAT1, pSTAT3 (Tyr705), STAT3, nephrin and podocin were analyzed by western blot **(C)**. **(D, E)** Mouse primary podocytes were treated with 10 ng/mL IL-6 for 24 hours **(D)** or 30 minutes **(E)** with or without baricitinib. Representative confocal microscopic images of mouse primary podocytes **(D)**. Cells were stained with DAPI (blue), fluorescence-conjugated antibodies to nephrin (green) and phalloidin dye (red) to identify F-actin. Expression levels of pJAK2, JAK2, pSTAT3 (Tyr705), STAT3, pSTAT1 and STAT1 were analyzed by western blot **(E)**. Data are expressed as mean ± SD. *p < 0.05; **p < 0.01; ***p < 0.001.

Previously, we have demonstrated that baricitinib could restore expression levels of functional proteins in podocytes and their mRNA levels in murine kidneys. These proteins are essential for maintaining cytoskeletal structures of podocytes and glomerular filtration functions (21). Therefore, we investigated whether baricitinib treatment could restore the actin cytoskeleton of podocytes disrupted by inflammation. Immortalized murine podocyte cell line in permissive condition was stimulated by IFNs with or without baricitinib. Inflammatory stimulus induced disintegration of cytoskeletal structures of podocytes as shown in confocal microscopic images. However, baricitinib treatment recovered it (**Figure 6A**). Expression levels of IFN-stimulated genes (ISGs) and intracellular proteins related to the JAK/STAT pathway including phosphorylated JAK2 (pJAK2), pSTAT1, and pSTAT3_Y705_ in differentiated podocytes were significantly down-regulated by treatment with baricitinib, whereas those of functional and structural proteins such as nephrin and podocin were increased by baricitinib (**Figures 6B, C**).

In addition to immortalized podocytes, primary podocytes were also isolated from glomeruli of C57BL/6 mice as described in Materials and Methods. These primary podocytes presented reduced expression of nephrin and distorted skeletal structures after IL-6 stimulation. However, such morphological abnormalities were ameliorated by treatment with baricitinib (**Figure 6D**). Furthermore, baricitinib treatment effectively suppressed phosphorylation of JAK2, STAT3, and STAT1 in these primary podocytes in a dose-dependent manner (**Figure 6E**). These *in vitro* results suggest that baricitinib could stabilize structural abnormalities of podocytes caused by inflammation related to the JAK/STAT pathway.

## Discussion

In the present study, baricitinib effectively attenuated lupus-related phenotypes including renal inflammation and modulated the population of pathogenic or regulatory immune cells in MRL/*lpr* mice. Especially, B cell differentiation and consequent Ig production stimulated by pro-inflammatory conditions were significantly suppressed by baricitinib through pharmacological inhibition of the JAK/STAT pathway. Selective JAK1 and JAK2 inhibition by baricitinib also stabilized actin cytoskeletal structures and restored the expression of elemental proteins in podocytes, ultimately exerting its efficacy on proteinuria caused by LN.

Therapeutic effects of JAK/STAT inhibition on various immune cells have been delineated by multiple *in vitro* studies (24). Baricitinib could suppress the production of type I IFNs from plasmacytoid dendritic cells (pDCs) as well as subsequent activation of pDCs and other myeloid DCs (24). In this study, these effects were demonstrated by decreased levels of circulating IL-6 and BAFF in lupus-prone mice, primarily originating from activated DCs. This selective inhibitor for JAK1 and JAK2 is also known to inhibit the differentiation of CD4+ naïve T cells into Th1 or Th17 cells, which possibly contributes to the pathogenesis of SLE (24). The present study did not present changes in the number of CD4+ T cells. Rather, it showed decreased populations of CD8+ T cells and DNT cells known to contribute to disease pathogenesis and activities in lupus (25). Furthermore, remarkably decreased proportions of central memory CD8+ T cells and increased ratios of Treg cells and Tfr cells were observed in mice treated with baricitinib. Although the exact role of central memory CD8+ T cells has not been sufficiently investigated in SLE (26), a few studies have reported that this T cell subset could be induced in the setting of autoimmunity due to stimulation by multiple cytokines, such as IL-2, IL-12, IL-15, IL-21, and type I IFNs, all of which are mediated *via* the JAK/STAT pathway (27). Increased proportions of T cells with regulatory function including Treg cells and Tfr cells could attenuate abnormal GC responses activated by autoreactive immune cells, consequently resulting in the secretion of autoantibodies with high affinities.

Differentiation and maturation of naïve B cells into autoantibody-secreting cells require stimulation of cytokines including IFNs and IL-6 (28–30). They also require interactions with antigen-presenting cells and helper T cells. Because signals of these cytokines are transduced through the JAK/STAT pathway, JAK inhibitors are expected to be effective in regulating B cell differentiation and Ig production. In this context, Wang et al. have reported that tofacitinib can inhibit B cell activation and differentiation (31). Kubo et al. have demonstrated that baricitinib could exert similar effects in *in vitro* studies (24). These results were similar to results of the present study, showing decreased expression levels of genes related to B cell maturation and consequent suppression of IgG production after treatment with baricitinib. Baricitinib significantly suppressed phosphorylation of STAT1 and STAT3 downstream of the JAK/STAT pathway in B cells both *in vitro* and *in vivo*. In addition to their antibody-secreting functions, auto-reactive B cells could act as sources of pro-inflammatory cytokines (29). They also play an antigen-presenting role to activate autoreactive T cells. Considering the profound role of B cells in the pathogenesis of SLE, there have been many therapeutic approaches targeting B cells, such as using anti-CD20 antibodies and anti-B-lymphocyte stimulator antibodies (32). Although results of clinical trials using these agents were disappointing or modest regarding their overall efficacy for SLE, JAK inhibitors including tofacitinib and baricitinib could be used as promising candidates for regulating aberrant activation of auto-reactive B cells in SLE according to previous studies and present results.

Podocytes are specialized epithelial cells consisting renal glomerulus. They are closely related to various clinical phenotypes caused by renal inflammation. However, the exact role of these cells in LN has not been fully investigated yet. Moreover, studies particularly focusing on the JAK/STAT pathway of podocytes in such autoimmune conditions have not been reported. Since previous studies on other chronic kidney diseases have demonstrated that JAK2 is overexpressed in diabetic renal diseases (33) and that podocyte loss is induced by type I IFN in viral glomerulonephritis (34), it is not an illogical leap that chronic autoimmune inflammation in patients’ kidneys with LN can promote persistent activation of the JAK/STAT pathway in podocytes. In LN, podocytes can induce and respond to inflammatory signals (21). That is, these cells can secrete pro-inflammatory cytokines such as IL-6 and tumor necrosis factor-alpha. In the meantime, they can be affected by such signals secreted by them. Under an inflammatory environment, expression levels of functional membranous proteins composing slit diaphragm are decreased, leading to structural changes of actin cytoskeleton of podocytes (21). Subsequently, these changes can cause foot process effacement of podocytes, thereby inducing clinical manifestations including proteinuria in LN (21). In the present study, we demonstrated that baricitinib could suppress the JAK/STAT pathway in podocytes, restore structural abnormalities induced by inflammatory conditions, and consequently prevent renal injuries in lupus-prone mice. To the best of our knowledge, this is the first report suggesting that JAK inhibitors could be efficacious for podocyte abnormalities in SLE.

Collectively, the present study demonstrated that baricitinib, a selective inhibitor for JAK1 and JAK2, could mitigate autoimmune features in lupus-prone mice by suppressing eccentric B cell activation and recovering structural injuries of podocytes. Although multiple novel JAK inhibitors with increased JAK selectivity have been developed, baricitinib is one of the most efficacious and safety-verified agents in large-scale clinical trials and post-market surveillance to date. Although the recently reported phase 2 trial and the currently ongoing phase 3 trial of baricitinib have focused on non-renal SLE patients, considering the role of the JAK/STAT pathway associated with SLE pathogenesis, baricitinib and more selective novel JAK inhibitors could be applied to SLE patients with other clinical manifestations including renal involvements based on results of this study.

## Data Availability Statement

The raw data supporting the conclusions of this article will be made available by the authors, without undue reservation.

## Ethics Statement

The animal study was reviewed and approved by the Institutional Animal Care and Use Committee (IACUC) of the School of Medicine, The Catholic University of Korea (approval numbers: CUMS-2018-0341-01 and 2018-0236-01).

## Author Contributions

JL and S-KK contrived all experiments. JL, SJ, S-MH, Y-SS, M-JK, and SB conducted experiments. JL, YP, and S-KK analyzed the data. JL, YP, and S-KK drafted and revised the manuscript. All authors contributed to the article and approved the submitted version.

## Funding

This work was supported by the National Research Foundation of Korea (NRF) grant funded by the Korea government (MSIT) (No. NRF-2018R1A2A2A05018848) and a grant of the Korea Health Technology R&D Project through the Korea Health Industry Development Institute (KHIDI), funded by the Ministry of Health & Welfare, Republic of Korea (grant number HI20C1496).

## Conflict of Interest

The authors declare that the research was conducted in the absence of any commercial or financial relationships that could be construed as a potential conflict of interest.

## Publisher’s Note

All claims expressed in this article are solely those of the authors and do not necessarily represent those of their affiliated organizations, or those of the publisher, the editors and the reviewers. Any product that may be evaluated in this article, or claim that may be made by its manufacturer, is not guaranteed or endorsed by the publisher.

## References

[1] Villarino AV Kanno Y O’Shea JJ. Mechanisms and Consequences of Jak-STAT Signaling in the Immune System. Nat Immunol (2017) 18:374–84. 10.1038/ni.3691 PMC11565648 28323260

[2] Zarrin AA Bao K Lupardus P Vucic D. Kinase Inhibition in Autoimmunity and Inflammation. Nat Rev Drug Discov (2021) 20:39–63. 10.1038/s41573-020-0082-8 33077936 PMC7569567

[3] Schwartz DM Kanno Y Villarino A Ward M Gadina M O’Shea JJ. JAK Inhibition as a Therapeutic Strategy for Immune and Inflammatory Diseases. Nat Rev Drug Discov (2017) 16:843–62. 10.1038/nrd.2017.201 29104284

[4] Kaul A Gordon C Crow MK Touma Z Urowitz MB van Vollenhoven R. Systemic Lupus Erythematosus. Nat Rev Dis Primers (2016) 2:16039. 10.1038/nrdp.2016.39 27306639

[5] Anders HJ Saxena R Zhao MH Parodis I Salmon JE Mohan C. Lupus Nephritis. Nat Rev Dis Primers (2020) 6:7. 10.1038/s41572-019-0141-9 31974366

[6] Banchereau J Pascual V. Type I Interferon in Systemic Lupus Erythematosus and Other Autoimmune Diseases. Immunity (2006) 25:383–92. 10.1016/j.immuni.2006.08.010 16979570

[7] Crow MK. Type I Interferon in the Pathogenesis of Lupus. J Immunol (2014) 192:5459–68. 10.4049/jimmunol.1002795 PMC4083591 24907379

[8] Kawasaki M Fujishiro M Yamaguchi A Nozawa K Kaneko H Takasaki Y. Possible Role of the JAK/STAT Pathways in the Regulation of T Cell-Interferon Related Genes in Systemic Lupus Erythematosus. Lupus (2011) 20:1231–9. 10.1177/0961203311409963 21980035

[9] Alunno A Padjen I Fanouriakis A Boumpas DT. Pathogenic and Therapeutic Relevance of JAK/STAT Signaling in Systemic Lupus Erythematosus: Integration of Distinct Inflammatory Pathways and the Prospect of Their Inhibition With an Oral Agent. Cells (2019) 8(8):898. 10.3390/cells8080898 PMC6721755 31443172

[10] Mok CC. The Jakinibs in Systemic Lupus Erythematosus: Progress and Prospects. Expert Opin Investig Drugs (2019) 28:85–92. 10.1080/13543784.2019.1551358 30462559

[11] Furumoto Y Smith CK Blanco L Zhao W Brooks SR Thacker SG. Tofacitinib Ameliorates Murine Lupus and Its Associated Vascular Dysfunction. Arthritis Rheumatol (2017) 69:148–60. 10.1002/art.39818 PMC5195893 27429362

[12] Wallace DJ Furie RA Tanaka Y Kalunian KC Mosca M Petri MA. Baricitinib for Systemic Lupus Erythematosus: A Double-Blind, Randomised, Placebo-Controlled, Phase 2 Trial. Lancet (2018) 392:222–31. 10.1016/s0140-6736(18)31363-1 30043749

[13] Fragoulis GE McInnes IB Siebert S. JAK-Inhibitors. New Players in the Field of Immune-Mediated Diseases, Beyond Rheumatoid Arthritis. Rheumatol (Oxf) (2019) 58:i43–54. 10.1093/rheumatology/key276 PMC6390879 30806709

[14] Fetter T Smith P Guel T Braegelmann C Bieber T Wenzel J. Selective Janus Kinase 1 Inhibition Is a Promising Therapeutic Approach for Lupus Erythematosus Skin Lesions. Front Immunol (2020) 11:344. 10.3389/fimmu.2020.00344 32194562 PMC7064060

[15] Maeshima K Shibata H. Efficacy of JAK 1/2 Inhibition in the Treatment of Diffuse Non-Scarring Alopecia Due to Systemic Lupus Erythematosus. Ann Rheum Dis (2020) 79:674–5. 10.1136/annrheumdis-2019-216571 31900301

[16] Kikawada E Lenda DM Kelley VR. IL-12 Deficiency in MRL-Fas(lpr) Mice Delays Nephritis and Intrarenal IFN-Gamma Expression, and Diminishes Systemic Pathology. J Immunol (2003) 170:3915–25. 10.4049/jimmunol.170.7.3915 12646661

[17] Theofilopoulos AN Dixon FJ. Murine Models of Systemic Lupus Erythematosus. Adv Immunol (1985) 37:269–390. 10.1016/s0065-2776(08)60342-9 3890479

[18] Richard ML Gilkeson G. Mouse Models of Lupus: What They Tell Us and What They Don’t. Lupus Sci Med (2018) 5:e000199. 10.1136/lupus-2016-000199 29387435 PMC5786947

[19] Crispín JC Oukka M Bayliss G Cohen RA Van Beek CA Stillman IE. Expanded Double Negative T Cells in Patients With Systemic Lupus Erythematosus Produce IL-17 and Infiltrate the Kidneys. J Immunol (2008) 181:8761–6. 10.4049/jimmunol.181.12.8761 PMC2596652 19050297

[20] Yang MH Suen JL Li SL Chiang BL. Identification of T-Cell Epitopes on U1A Protein in MRL/lpr Mice: Double-Negative T Cells Are the Major Responsive Cells. Immunology (2005) 115:279–86. 10.1111/j.1365-2567.2005.02139.x PMC1782149 15885135

[21] Wright RD Beresford MW. Podocytes Contribute, and Respond, to the Inflammatory Environment in Lupus Nephritis. Am J Physiol Renal Physiol (2018) 315:F1683–94. 10.1152/ajprenal.00512.2017 PMC6336988 30207171

[22] Ozaki K Spolski R Ettinger R Kim HP Wang G Qi CF. Regulation of B Cell Differentiation and Plasma Cell Generation by IL-21, A Novel Inducer of Blimp-1 and Bcl-6. J Immunol (2004) 173:5361–71. 10.4049/jimmunol.173.9.5361 15494482

[23] Zotos D Coquet JM Zhang Y Light A D’Costa K Kallies A. IL-21 Regulates Germinal Center B Cell Differentiation and Proliferation Through a B Cell-Intrinsic Mechanism. J Exp Med (2010) 207:365–78. 10.1084/jem.20091777 PMC2822601 20142430

[24] Kubo S Nakayamada S Sakata K Kitanaga Y Ma X Lee S. Janus Kinase Inhibitor Baricitinib Modulates Human Innate and Adaptive Immune System. Front Immunol (2018) 9:1510. 10.3389/fimmu.2018.01510 30002661 PMC6031708

[25] Alexander JJ Jacob A Chang A Quigg RJ Jarvis JN. Double Negative T Cells, a Potential Biomarker for Systemic Lupus Erythematosus. Precis Clin Med (2020) 3:34–43. 10.1093/pcmedi/pbaa001 32257532 PMC7093895

[26] Sen Y Chunsong H Baojun H Linjie Z Qun L San J. Aberration of CCR7 CD8 Memory T Cells From Patients With Systemic Lupus Erythematosus: An Inducer of T Helper Type 2 Bias of CD4 T Cells. Immunology (2004) 112:274–89. 10.1111/j.1365-2567.2004.01862.x PMC1782491 15147571

[27] Raeber ME Zurbuchen Y Impellizzieri D Boyman O. The Role of Cytokines in T-Cell Memory in Health and Disease. Immunol Rev (2018) 283:176–93. 10.1111/imr.12644 29664568

[28] Domeier PP Chodisetti SB Schell SL Kawasawa YI Fasnacht MJ Soni C. B-Cell-Intrinsic Type 1 Interferon Signaling Is Crucial for Loss of Tolerance and the Development of Autoreactive B Cells. Cell Rep (2018) 24:406–18. 10.1016/j.celrep.2018.06.046 PMC6089613 29996101

[29] Ma K Du W Wang X Yuan S Cai X Liu D. Multiple Functions of B Cells in the Pathogenesis of Systemic Lupus Erythematosus. Int J Mol Sci (2019) 20(23):6021. 10.3390/ijms20236021 PMC6929160 31795353

[30] Do-Thi VA Lee JO Lee H Kim YS. Crosstalk Between the Producers and Immune Targets of IL-9. Immune Netw (2020) 20:e45. 10.4110/in.2020.20.e45 33425430 PMC7779872

[31] Wang SP Iwata S Nakayamada S Sakata K Yamaoka K Tanaka Y. Tofacitinib, A JAK Inhibitor, Inhibits Human B Cell Activation. Vitro Ann Rheum Dis (2014) 73:2213–5. 10.1136/annrheumdis-2014-205615 25157177

[32] Chan VS Tsang HH Tam RC Lu L Lau CS. B-Cell-Targeted Therapies in Systemic Lupus Erythematosus. Cell Mol Immunol (2013) 10:133–42. 10.1038/cmi.2012.64 PMC4003049 23455017

[33] Zhang H Nair V Saha J Atkins KB Hodgin JB Saunders TL. Podocyte-Specific JAK2 Overexpression Worsens Diabetic Kidney Disease in Mice. Kidney Int (2017) 92:909–21. 10.1016/j.kint.2017.03.027 PMC5610635 28554737

[34] Migliorini A Angelotti ML Mulay SR Kulkarni OO Demleitner J Dietrich A. The Antiviral Cytokines IFN-α and IFN-β Modulate Parietal Epithelial Cells and Promote Podocyte Loss: Implications for IFN Toxicity, Viral Glomerulonephritis, and Glomerular Regeneration. Am J Pathol (2013) 183:431–40. 10.1016/j.ajpath.2013.04.017 23747509

