# Supplementary material for: Baricitinib Attenuates Autoimmune Phenotype and Podocyte Injury in a Murine Model of Systemic Lupus Erythematosus
Source: Front Immunol. 2021 Aug 23;12:704526. doi: 10.3389/fimmu.2021.704526 (PMC8419414; doi:10.3389/fimmu.2021.704526)

**<Supplementary Materials>**

**Supplementary Figure 1.** Representative images showing gross appearance of spleens (the upper panels) and cervical lymph nodes (the lower panels) from 8-week-old MRL/*lpr* mice.


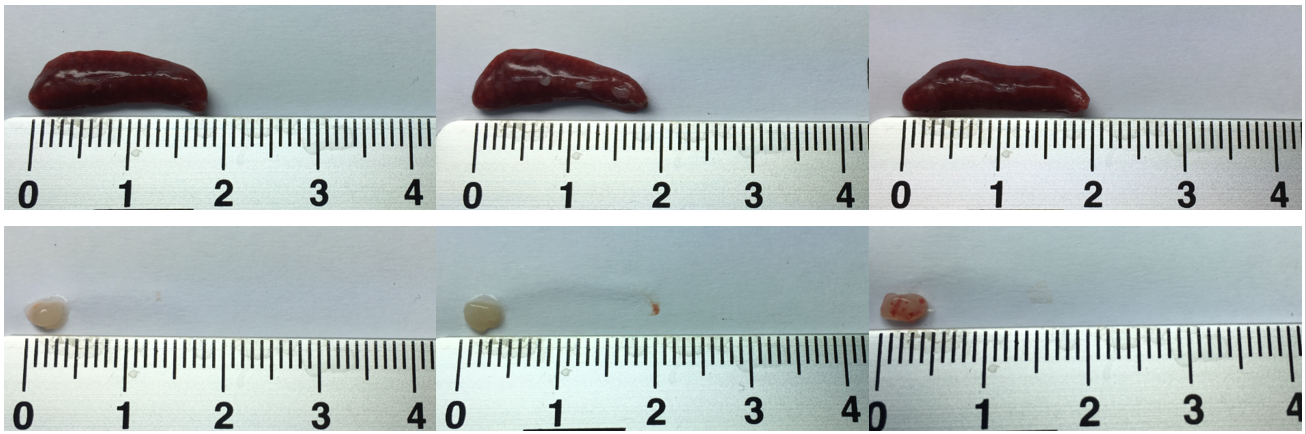


**Supplementary Figure 2.** The gating strategies of flow cytometry analysis in **Figure 2B**. The gating strategy for central memory CD8+ T cells (CD44+ CD62L+) is as lymphocytes in forward scatter (FSC)-A vs. side scatter (SSC)-A, singlets in FSC-H vs. FSC-A and live cells in fixable viability dye-A vs. FSC-A.


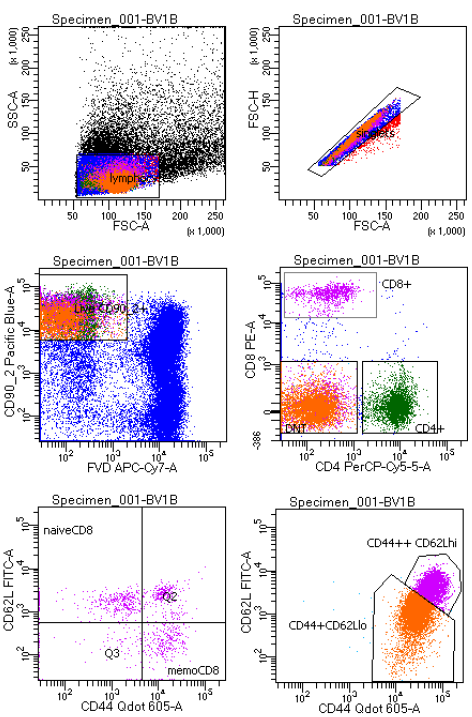


**Supplementary Figure 3.** Representative dotplots of regulatory T cells (CD4+CD25+CXCR5-FOXP3+, the upper panels), follicular regulatory T cells (CD4+CD25-CXCR5+PD-1+FOXP3+, the lower panels), and follicular helper T cells (CD4+CD25-CXCR5+PD-1+FOXP3-, the lower panels) in **Figure 2C**.


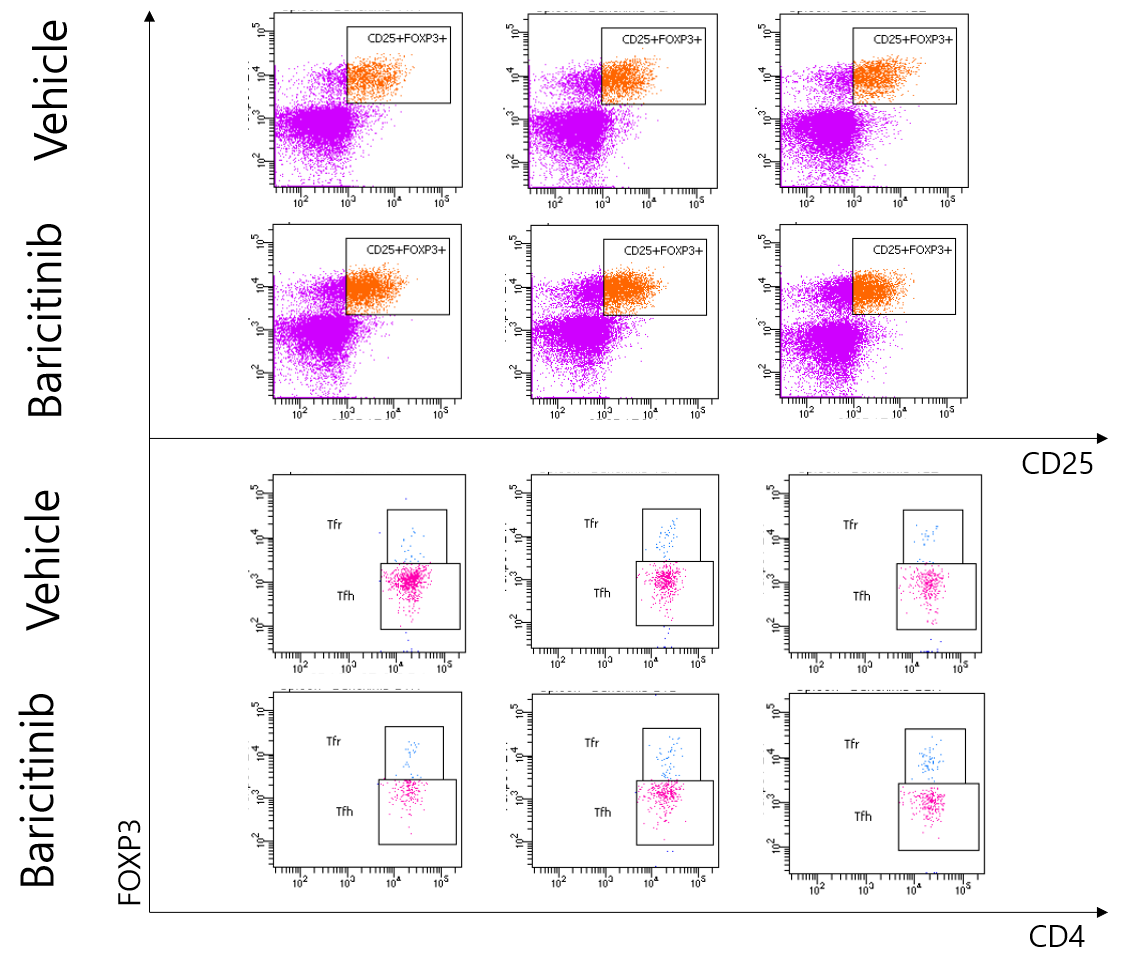


**Supplementary Figure 4.** Representative dotplots of regulatory T cells (CD4+CD25+CXCR5- FOXP3+) in **Figure 3E**.


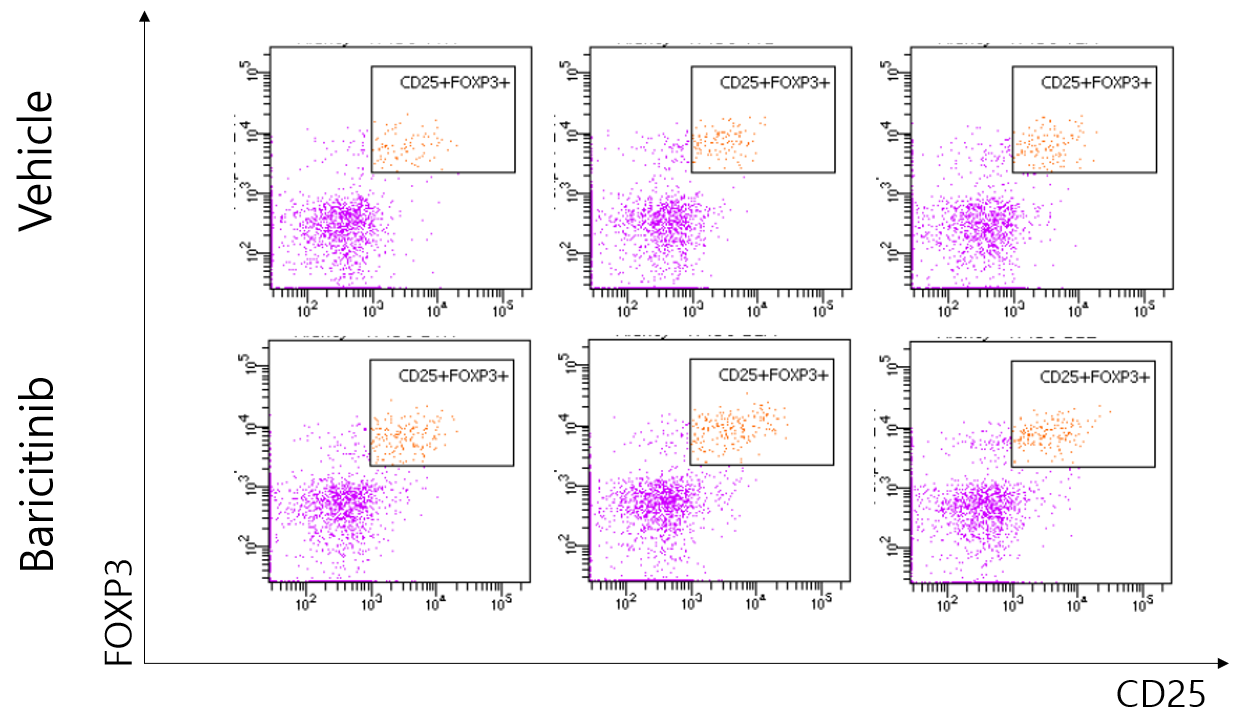


**Supplementary Figure 5.** **(A)** Representative flow cytometric image showing CD19+ B cells isolated from spleens of MRL/*lpr* mice. **(B)** B cells (5 × 10^5^ cells per well) treated with 1 μg/mL IgM antibodies, 250 ng/mL sCD40L, and 100 ng/mL IL-4 for 2 days with or without graded dose of baricitinib. mRNA levels of *Aicda*, and *Bcl6* were determined using real-time PCR. **(C)** B cells were treated with the same condition for 5 days. mRNA levels of *Xbp1* and *Irf4* were determined using real-time PCR. **(D)** Levels of IgG in culture supernatant were measured by ELISA.


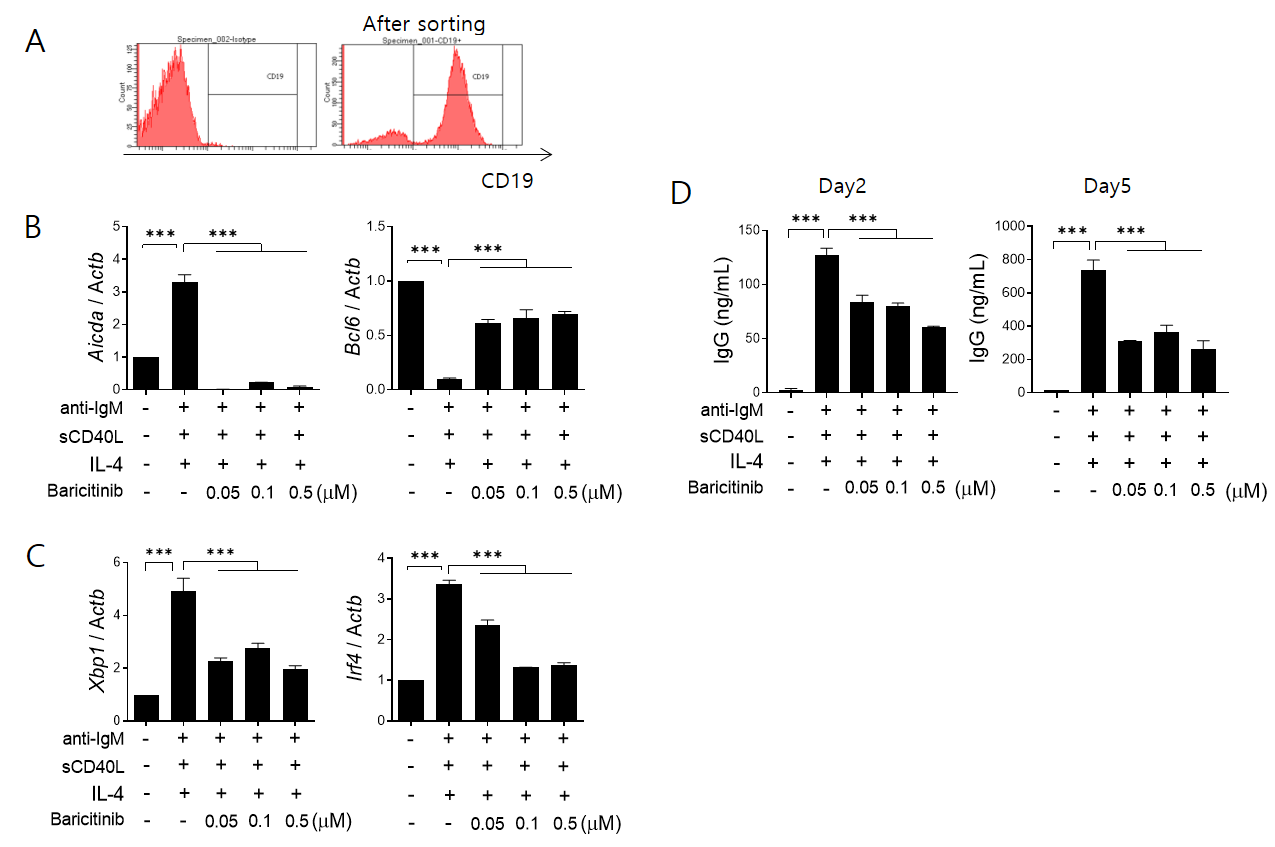

Supplement: Supplementary file 1 [file DataSheet_1.docx]
